# Perceived Parental Rejection Mediates the Influence of Serotonin Transporter Gene (5-HTTLPR) Polymorphisms on Impulsivity in Japanese Adults

**DOI:** 10.1371/journal.pone.0047608

**Published:** 2012-10-24

**Authors:** Saori Nishikawa, Shota Nishitani, Takashi X. Fujisawa, Ippei Noborimoto, Takayuki Kitahara, Tsunehiko Takamura, Kazuyuki Shinohara

**Affiliations:** 1 Department of Neurobiology and Behavior, Graduate School of Biomedical Sciences, Nagasaki University, Nagasaki, Japan; 2 Phoenix Leader Education Program, Hiroshima University, Higashi Hiroshima, Japan; Institut Jacques Monod, France

## Abstract

This study examined (1) the interrelationships among 5-HTTLPR genotype, perceived parental rejection, and impulsivity, and (2) meditational models in which perceived paternal/maternal rejection mediates the relationship between the 5-HTTLPR genotype and impulsive behaviour. Participants included 403 adults (152 males and 252 females, mean age = 24.20) who provided genetic data and a set of the questionnaires (BIS11; Barratt Impulsiveness Scale-11 and EMBU; Egna Minnen av Bätraffande Uppfostran). Using SEM (Structural Equation Modeling), we evaluated 3 models for both direct and indirect relationships between 5-HTTLPR (5HTT) and Impulsivity (IMP), via maternal/fraternal rejection (MAT/FAT). In model 1, the direct path from 5HTT and IMP was not significant across the mother’s and father’s analysis. Models 2 and 3 assessed the indirect influence of 5HTT on IMP through MOT/FAT. The paths of models 2 and 3 were all significant and showed a good fit between the hypothesized model and data. Furthermore, the effects of the 5-HTTLPR genotype on impulsiveness in this Japanese sample were particularly accounted for by perceived rejection from the mother or father. The effects from the parents appeared to be robust especially among males. These results may help elucidate the specific pathways of risk in relation to genetic and environment influences on impulsive phenotypes.

## Introduction

Violence is one of the international issues that researchers have set a task to prevent. There have been concerns about how we solve problems relating to violent, aggressive, and impulsive behaviour, such as DV, child maltreatment, neglect, and adolescent delinquency. Impulsivity is treated as one of the symptoms that cuts across psychiatric disorders, and social, biological, and psychological etiologies of treatment are necessary. Moeller et al [1] defined impulsivity as 1) decreased sensitivity to negative consequences of behaviour, 2) rapid, unplanned reactions to stimuli before complete processing of information, and 3) a lack of regard for long-term consequences.

The serotonin transporter (5HT) is a key regulator of serotonin neurotransmission. 5-HTTLPR (serotonin transporter linked polymorphic region) consists of two common alleles differing by 44 pairs, short (S) and long (L) variants, where the S allele has lower transcription efficiency than the L allele [2]. Serotonin plays a role in biological processes (i.e. mood, food intake, sleep) and social behaviour. Some researchers examined the influence of early social interaction and 5-HTTLPR on psychological problems. Mothers with the 5-HTTLPR SS genotype were less sensitive than mothers with SL or LL [3]. It was shown that a stable loneliness among the S allele, and especially S carriers who perceived little support from their mothers, were at the risk of developing a loneliness [4]. Some studies have suggested that parenting style influences the development of impulsiveness in S carriers [5], [6], [7].

It is now well known that early experiences with a caregiver influence psychological development in later life (e.g. [8]), as it is seen in a social context, i.e. parental attachment and caregiving [9], [10], and mating behaviour [11], [12]. It has been reported that emotional over involved parental behaviour has a negative influence on adolescent internalizing problems (depression and anxiety), and that gender, type of parenting, and the developmental period plays a role in these associations. Fox et al [13] reported a 5HTT - environment interaction in children, with S carriers and low social support showing an increased risk for behavioural inhibition.

Research into gene - environmental interactions is relatively new in the field of psychiatry, psychology, and neuroscience. It has been generally accepted that genetics and the environment contribute to personality and behaviour. However, subsequent studies of gene -environment interactions at 5-HTTLPR and personality or behaviour are still inconsistent. Munafo et al [14] developed the meditational model Neuroticism (i.e. anxiety related personality traits) as a mediator of the association between 5-HTTLPR genotype and major depression, as well as a straight forward association between 5-HTTLPR, neurosis, anxiety, and depression [15]. However, more recently, it has been reported that 5-HTTLPR is not associated with the Harm Avoidance personality [16]. Using a widely used self-report measure (BIS-11; Baratt Impulsive Scale-11 [17]), it was shown that individuals with a homozygous S-allele were more impulsive (Attentional Impulsivity and Total Impulsivity) than individuals with the other genotype [18]. On the other hand, other studies [19], [20] found no associations between impulsivity measured by BIS-11 and the 5-HTTLPR genotype.

The serotonergic system is said to behave differently depending on gender. Some studies suggested gender differences in the effects of 5-HTTLPR on behaviour. It was shown that females with the S allele and males with the L allele were at the highest psychological risk [21], [22]. Munafo et al [23] suggested gender differences in the association between 5-HTTLPR genotypes and alcohol consumption. They showed that the S allele is associated with increased alcohol consumption among men. In an Italian sample, it was shown that women had a higher rating of motor impulsivity, cognitive complexity, and total score of BIS11 [24], whereas the 5-HTTLPR system was more involved in impulsive behaviour among men than women. A relationship between 5-HTTLPR and the risk of impulsive-disinherited personality among male offenders has also been reported [25].

In summary, genotype/personality studies are notoriously difficult to interpret, and their results are often controversial. Despite early studies suggesting the 5-HTTLPR genotype as a player in social behaviour, neurobiological factors contributing to behavioural problems in humans remain poorly understood. A few studies have reported the influence of the serotonin system on impulsive behaviour and perceived parental rearing in a non-referred sample. Furthermore, we sought to address the question of whether perceived fraternal/maternal rejection mediates 5-HTTLPR and impulsive behaviour in adults drawn from the general population. Therefore, the aims of the present study were to: (1) examine the interrelationships among 5-HTTLPR, perceived parental rejection, and impulsivity, and (2) test a meditational model in which perceived paternal/maternal rejection mediates the relationship between the 5-HTTLPR genotype and impulsive behaviour. In the light of findings reported about associations between the serotoninergic system and social behaviour, we hypothesize that the relationship between the 5-HTTLPR genotype and impulsive behaviour is mediated by rejection from the mother or father.

## Methods

### Subjects

Participants for the present study included 403 individuals who provided both genetic data and questionnaires (152 males and 252 females, range = 18–49, mean age = 24.20, SD = 6.12), from a total sample of 741 adults who provided a set of questionnaires at the University (Faculty of Medicine, Education, Engineering) and Vocational College in Nagasaki city (391 males and 350 females, range = 18–51, mean age = 23.63, SD = 5.74).

### Procedures

The study protocol was approved by Nagasaki University, and all participants provided written informed consent after receiving a full explanation of the study. Questionnaires and saliva sample collection containers were administrated during lectures. In addition, students and staff distributed questionnaires to their friends and neighbours in order to obtain a more diverse range of ages. After an explanation of the present study, participants provided informed consent, a saliva sample for DNA analyses, and a series of self-report measures of demographics, perceived parenting, and impulsive behaviour. It took approximately 30–40 minutes for the whole procedure. A saliva sample was not collected from all participants due to the class schedule. From a total sample of 741 adults who provided the questionnaires, data from 403 individuals who provided saliva samples and questionnaires were used for the present study.

### Instruments

#### Barratt impulsiveness scale-11 (BIS11)

BIS11 [17] is a 30-item questionnaire with a 4-point Likert-type scale (1-rarely, 2-sometimes, 3-often, 4-almost always) to measure impulsivity divided in three subscales: (a) Attentional Impulsivity, (b) Motor Impulsivity, and (c) Non-planning Impulsivity. A Japanese version of BIS11 has been developed [26], and it has shown good psychometric properties with Cronbach’s alpha ranging from 0.64 to 0.65 (mean = 0.63), with a total BIS11 score of 0.80.

#### EMBU (egna minnen av bätraffande uppfostran)

EMBU (Egna Minnen av Bätraffande Uppfostran – My memories of upbringing [27]) is a Swedish self-report measure of adult perceptions about the rearing behaviour of one's parents with subscales of Rejection, Emotional Warmth, Overprotection, and Favouring Subject. EMBU consists of 81 items on a 4-point Likert scale (1–4). The Japanese version was developed, and it has shown good psychometric properties [28].

### Genotyping

Genomic DNA was extracted from oral mucosa collected from participants using the QIAamp DNA Micro Kit (QIAGEN, Tokyo, Japan). This study was performed using ethnically homogeneous individuals (only of Japanese descent). The serotonin transporter gene (5-HTTLPR) of the 5-HTT gene regulatory region was amplified by polymerase chain reaction (PCR) with a forward primer (5′-GGCGTTGCCGCTCTGAATGC-3′) and reverse primer (5′-GAGGGACTGAGCTGGACAACCAC-3′). For PCR, 10 ng of genomic DNA was used in a 25-µL reaction mixture containing 0.5 U of KOD FX Neo (Toyobo Co., LTD.) and 10 pmoles of each primer in PCR buffer for KOD FX Neo (Toyobo Co., LTD.). Cycling conditions were as follows: denaturation (94°C for 2 min) and 30 cycles of amplification (98°C for 10 sec, 63°C for 30 sec, and 68°C for 30 sec). PCR products were separated by electrophoresis in a 3% agarose gel and visualized by UV after ethidium bromide staining. A 484 bp band was observed for the S allele, and a 528 bp band for the L allele; heterozygous samples showed both alleles. Two investigators scored allele sizes independently and any inconsistencies were reviewed and rerun.

**Table 1:** Correlations between factors.

	1	2	3	4	5	6	7	8
1 Rejection Father	―							
2 Rejection Mother	.59**	―						
3 Attentional Imp	.08	.15*	―					
4 Motor Imp	.17**	.18**	.54**	―				
5 Non planning Imp	.07	.05	.40**	.39**	―			
6 Total Imp	.14*	.15**	.79**	.83**	.75**	―		
7 5HTT	.06	.09	.12	−.01	.05	.02	―	
8 Gender	.13*	.06	−.04	−.03	−.10	−.05	−.03	―

Note;

** *p*<.001 *p<.05.

**Figure 1:**
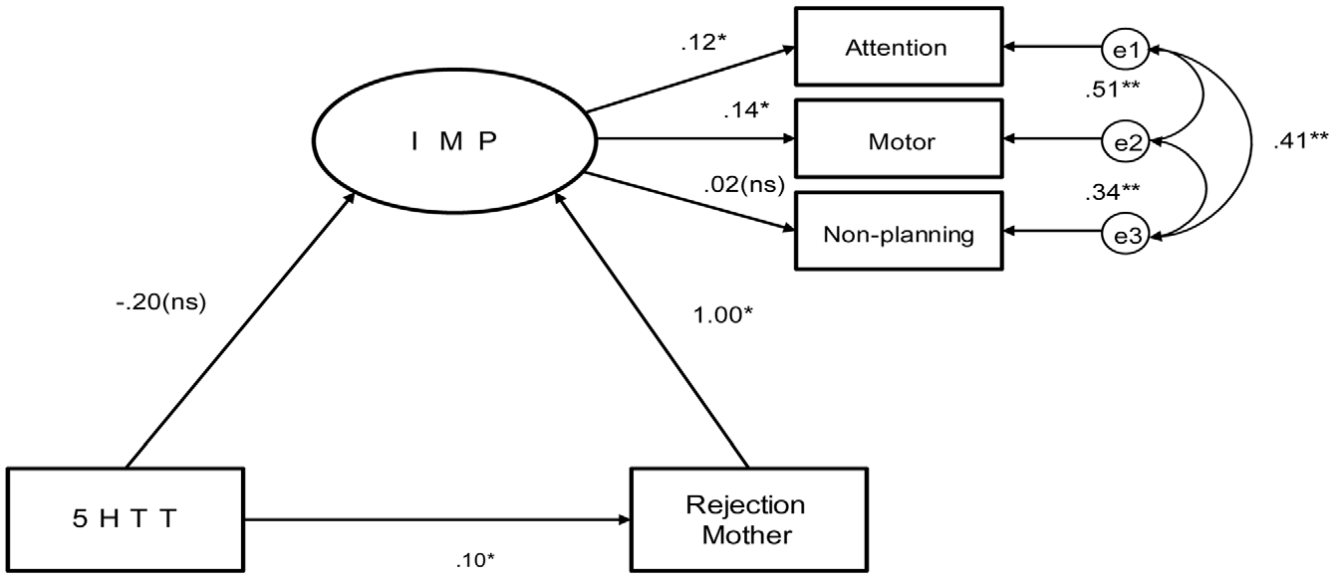
Model 1 (Mother). Note: All paths significant (** *p<*.001, * *p<*.05) unless indicated (ns). X^2^ (df2) = 11.63; CMIN/DF = 0.41;CMIN = 0.83 GFI = 1.00; RMSEA = 0.00.

### Data Analysis

SPSS (The Statistical Package for Social Sciences) version 17.0 [29] was used for computing descriptive statistics, correlations, and ANOVAs (analysis of variance). SEM (Structural Equation Models) were analysed using AMOS 16 on SPSS [29] to evaluate the mediating effects of the variables specified by models with path diagrams. This software performs analyses of moment structures through maximum likelihood estimation. To investigate whether a variable X (Rejection Father/Mother) is a mediator between independent variable A (5HTT) and dependent variable B (IMP) in the path analysis, a direct path from A to B is drawn in a first analysis. In the next analysis, two paths are added, one from A to X and the other from X to B. If X is a significant mediator, the weight of the path from A to B will decrease in the second analysis in comparison to the first one [30].

The GFI (Goodness of Fit Index) is considered a reasonable statistic index for evaluating a model and assesses the fit between a hypothesized model and the data. AMOS calculates all measures that capture model evaluation that were selected based on different theoretical perspectives such as: CMIN/df (the minimum value of sample discrepancy divided by its degree of freedom, smaller values preferable [31]); GFI (The Goodness of Fit Index, a measure of the relative amount of variance and covariance, close to 1, over 0.9 is preferable [32]), and RMSEA (the root mean square error of approximation based on population discrepancy, smaller values, below 0.08 preferable[33]).

## Results

### Descriptive Analysis

Of the 403 participants, 273 (67.7%) were homozygous for the short allele, 111 (27.5%) carried the heterogeneous genotype, and 19 (4.7%) were homozygous for the long allele. 5-HTTLPR groups did not differ significantly in regard to gender. To test whether levels of maternal parenting differed from paternal rearing, we conducted a pair samples t-test. Results showed that ratings of mothers were significantly higher than ratings of fathers in regard to all EMBU scales; Rejection (*t* (322) = 2.20, *p<*.05), Overprotection (*t* (322) = -13.5, *p<*.001), and Emotional Warmth (*t* (322) = −10.65, *p<*.001).

**Table 2:** Comparisons among pathways from 5HTT to perceived rejection and impulsivity, using various measures of model fit.

Model	X^2^ (df)	CMIN/df	CMIN	GFI	RMSEA
Model 1 (All)	.08 (2)	.41	.83	1.00	.000
Model 2 (Mother)					
(All)	1.63 (4)	.46	1.84	1.00	.000
(Male)	2.45 (4)	.61	2.45	1.00	.000
(Female)	.71 (4)	.17	1.84	1.00	.000
Model 3 (Father)					
(All)	1.60 (4)	.40	1.60	1.00	.000
(Male)	2.13 (4)	.53	2.13	1.00	.000
(Female)	.68 (4)	.17	.684	1.00	.000

Regarding scales of Emotional Warmth, a univariate F-test showed a significant effect of maternal warmth on Non-planning, *F* (1,240) = 7.36, Eta^2^ = .03, *p*<.005, suggesting that individuals who perceived their mother as less loving tended to report more Non-planning Impulsivity than those who reported more maternal warmth. There was a gene -mother interaction on Motor Impulsivity, *F* (1,240) = 3.68, Eta^2^ = .025, *p*<.005 suggesting that SS carriers with low maternal warmth reported more Motor Impulsivity than SS carriers with high maternal warmth. Regarding Emotional Warmth Father, an effect of fraternal warmth was shown on Non- planning Impulsivity, *F* (1,234) = 5.98, Eta^2^ = .025, *p*<.005. There was a gene - father interaction on Attention Impulsivity, *F* (1,234) = 5.95, Eta^2^ = .025, *p*<.005, and Motor Impulsivity, *F* (1,234) = 5, Eta^2^ = .025, *p*<.005, suggesting that SS with low warmth from fathers tended to report more Attention and Motor Impulsivities than the SS type with more fraternal warmth.

**Figure 2:**
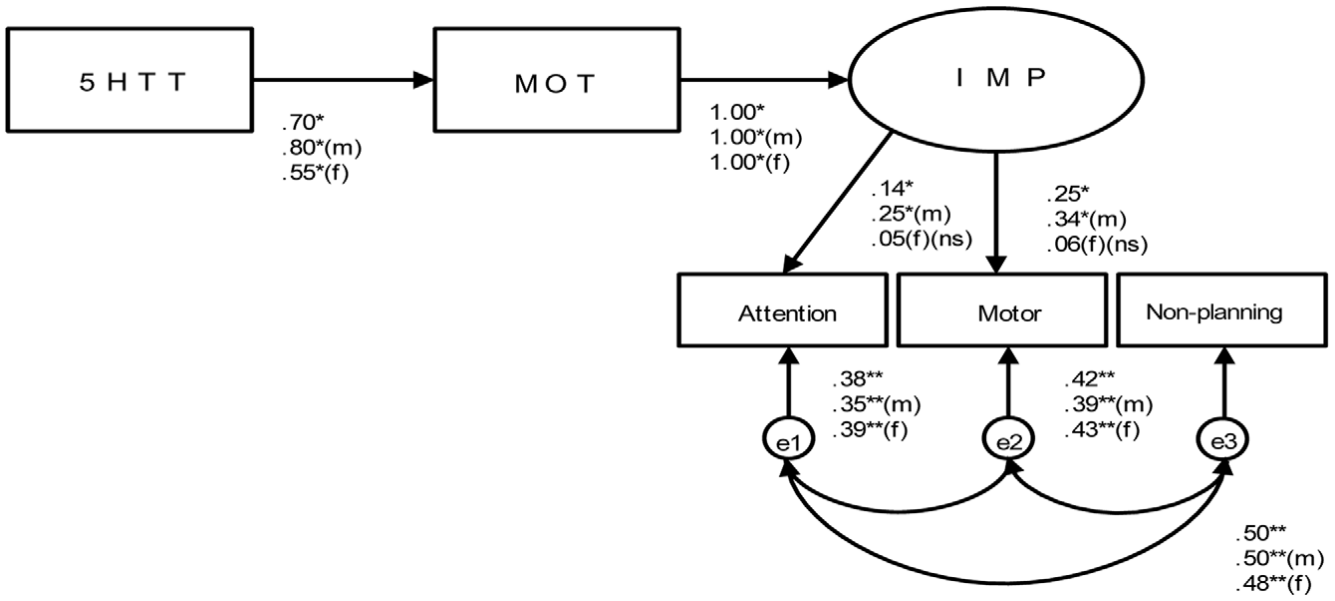
Model 2 (Mother). Note: All paths significant (** *p<*.001, * *p<*.05) unless indicated (ns). X^2^ (df4) = 1.63; CMIN/DF = 0.46; CMIN = 1.84; GFI = 1.00; RMSEA = 0.00 for all subjects included. (m) = only male, (f) = only female.

Regarding the scale of Rejection, a univariate F-test showed a significant effect of maternal rejection on Attention Impulsivity, *F* (1,223) = 4.24, Eta^2^ = .03, *p*<.005, Motor Impulsivity *F* (1,223) = 4.24, Eta^2^ = .03, *p*<.005, and Total Impulsivity *F* (1,223) = 4.24, Eta^2^ = .03, *p*<.005, indicating that individuals who perceived their mother as rejecting tended to report more Attention, Motor, and Total Impulsivities than those who reported less maternal rejection. There were no significant effects on genotype or gene - father interaction. There were no significant main effects on father’s rejection, genotype, or gene - father interaction.

**Figure 3:**
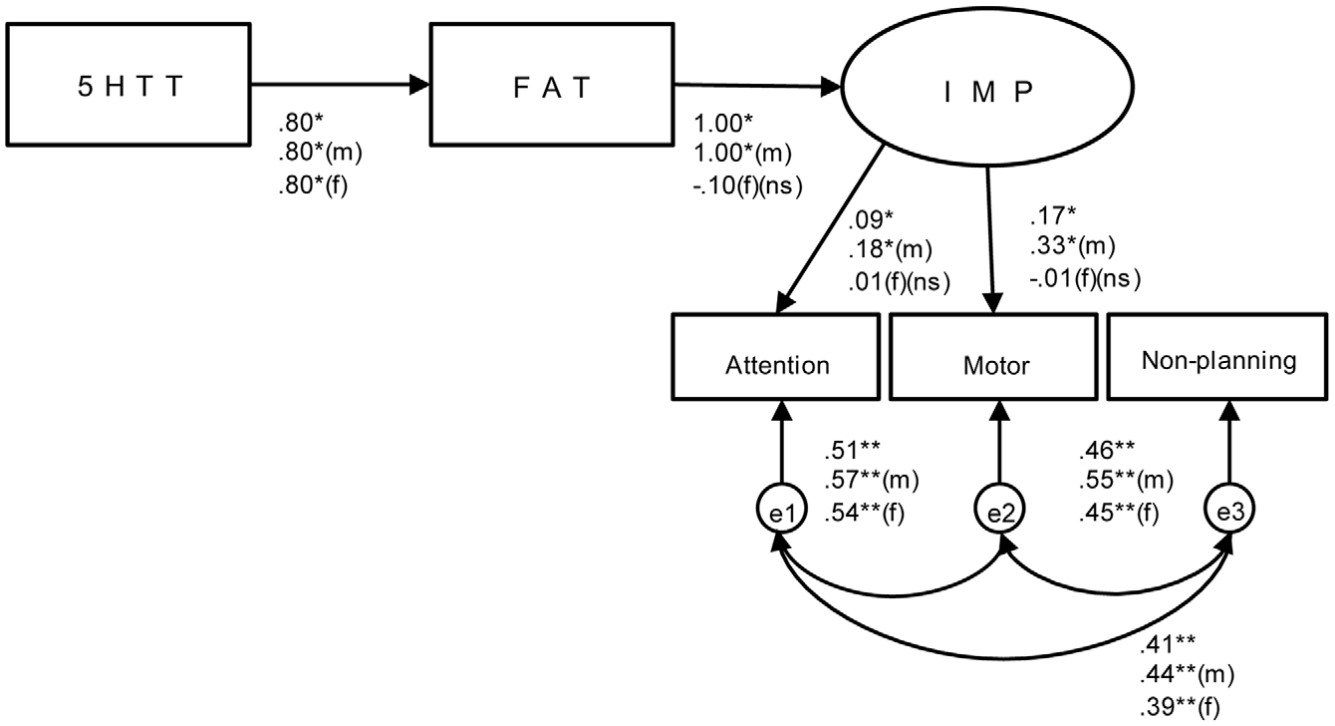
Model 3 (Father). Note: All paths significant (** *p<*.001, * *p<*.05) unless indicated (ns). X^2^ (df4) = 1.60; CMIN/DF = 0.40; CMIN = 1.60, GFI = 1.00; RMSEA = 0.00 for all subjects included. (m) = only male, (f) = only female.

### Evaluation of SEM

Before testing models, intercorrelations among factors were analysed. Correlations between the scales of BIS11, and perceived rejection and other variables are presented in Table 1. Attention Impulsivity was positively associated with Rejection Mother (*r = .*150, *p<*.001), but not with Rejection Father. Rejection from both the mother and father was associated with Motor Impulsivity (*r = .168* for father, *r = .*179 for mother, both *p<*.001) and Total Impulsivity (*r = *.139, *p<*.005 for father, *r = *.153, *p<*.001 for mother).

Figure 1 presents the standardized solution for SEM, specifying 5-HTTLPR (5HTT) as a direct determinant of perceived parental rejection from the father/mother and impulsivity. The estimated meditational model with standardized path coefficients is presented in Table 2. Following results of correlational analyses, subscales from BIS11 (Motor, Attention, Non-planning) and EMBU (Rejection Father/Mother) were used for the model.

The following strategy was used to test mediation. First, a model was estimated with only the predictor (i.e. 5HTT) and outcome variable (i.e. IMP) included, assessing the direct effect exclusive of any mediator variables. In the first model, both father and mother and genotype were included as a predictor in the model. Parent rejection (PAR) as mediating the relationship between 5HTT and IMP showed a relatively good fit (CMIN/df = .828, CMIN = 5.79, CFI = 1.00, RMSEA = .000), but the pathway did not evaluate a shared variance of variables for 5HTT, PAR, and IMP.

Second, we investigated the model analysis for mother and father separately, and included a direct path from 5HTT to IMP in the context of the meditational model to determine whether a significant bivariate relationship between predictor and outcome exists. Model 1 was explored in the model illustrated in Figure 1. The pathways linking 5HTT, PAR, and IMP were all significant (*p*>.05); however, the direct pathway from 5HTT to IMP was not significant when analysing neither the father’s nor mother’s rejection.

Third, the next possibility was explored in the model illustrated in Figure 2. Because the pathways between 5HTT and IMP were not significant in model 1, model 2 was evaluated, which eliminated this pathway. By using Rejection Mother (MOT), model 2 assessed the indirect influence of 5HTT on IMP through MOT. Pathways linking 5HTT with MOT with IMP were both significant (*p<*.001). The relationships between all constructs and their indicators were positive and significant (*p<*.001). Indices of goodness of fit indicated an acceptable fit between model 2 and the data (X^2^(4) = 1.63,CMIN/df = .399, CFI = 1.00, RMSEA = .000; see Table 2). Model 2 was also evaluated separately for males and females. All paths in model 2 were significant across gender (*p*<.001 and *p*<.05). Indices of goodness of fit showed acceptable fits: X^2^(4) = .2.45,CMIN/df = .171, CFI = 1.00, RMSEA = .000 for males, and X^2^(4) = .71,CMIN/df = 2.45, CFI = 1.00, RMSEA = .000 for females (see Table 2).

Finally, Figure 3 shows that model 3 using Rejection Father (FAT) was found to provide an adequate fit of the data, X^2^(4) = 1.60, CMIN/df = .40, CMIN = 1.84, CFI = 1.00, RMSEA = .000. This model reached an adequate fit across gender; X^2^(4) = 2.13,CMIN/df = .53, CMIN = 1.60, CFI = 1.00, RMSEA = .000 for males, and X^2^(4) = .68, CMIN/df = .17, CMIN = .684, CFI = 1.00, RMSEA = .000 for females. All paths (5HTT, FAT, IMP) were significant among males (*p*<.05), but not among females. Despite the significant path between 5HTT and FAT (*p*<.05), the path linking FAT and IMP was not significant among females.

## Discussion

The present study examined a meditational model in which perceived paternal/maternal rejection mediates the relationship between the 5-HTTLPR genotype and impulsive behaviour. The main result was that the effects of the 5-HTTLPR genotype on impulsiveness in this Japanese sample can be particularly accounted for by perceived parental rejection, especially from the mother. The effects from the parents appeared to be robust especially among males.

We evaluated two models regarding both direct and indirect relationships between 5-HTTLPR and impulsivity via perceived rejection. Model 1 provided a reasonable fit with the data. However, the direct path from 5HTT and IMP was not significant. This may reflect results from earlier studies [19], [20], which showed no effects of 5-HTTLPR on impulsivity. The path for Non Planning did not reflect a shared variance in IMP, as Non Planning, which reflects a lack of future orientation, and seems to have less influence on perceived parenting than that of other variables. This reflects results of correlational analyses showing no significant associations with Rejection Father or Mother. Using different areas of impulsive behaviours, it was possible to determine whether the model fit equally in explaining a broad range of problem behaviours.

The path of model 2 was all significant and showed a good fit between the hypothesized model and data across males and females. Perceived parenting formed as rejection from the father or mother interacted with genotype in relation to self-reported impulsive behaviours. This result is in line with results from studies that showed associations between the 5-HTTLPR genotype, low maternal care and loneliness [4], and observed parenting [3]. Impulsivity has been shown to be related to low serotonin turnover and a poor family environment [6].

Despite the power analysis, which showed no significant gender effect or gene - gender interaction, the result of SEM analysis showed a better model fit on rejection from both the mother and father among males than females. Similarly, rejection from the mother seems to play an important role in impulsivity as the model fit of the mother was better than rejection from the father. As seen in a comparison of the ratings for the mother and father on EMBU, it was shown that mother’s scores of all four factors (i.e., Emotional Warmth, Rejection, Overprotection, and Favouring Subject) were significantly higher than those rating fathers. It may reflect the traditional Japanese childrearing style where the mother is regarded as the one to take care of the children. However, results showed that it is necessary to consider not only the mother’s, but also the father’s rearing when analysing problem behaviour in addition to the genotype.

It is important to note the limitations of the present study. First, the present study was designed as cross-sectional and hence does not provide directions or causal effects. It is equally plausible that a higher degree of impulsivity was contributed to negative perceived parenting and genes, or impulsive individuals elicited negative evaluations from their surroundings. Second, generalization of our findings may be limited due to a low sample size because of the large number of excluded participants as a consequence of a lack of a DNA sample, and there were more females than males. Power analysis indicated in the present study may have been underpowered to detect effects or associations with 5-HTTLPR. It should also be mentioned that SL and LL genotypes were combined in the MANOVA analysis. Further study in a larger population from both clinical and non-clinical samples is needed to avoid stratification artifacts. Third, measurements are solely based on self-reports that involve a risk for information bias from recall and interpretation of past behaviour [34]. Future studies should include a behavioural laboratory to measure impulsivity. He et al [35] used two decision making tasks (Iowa Gambling Task and Loss Aversion Task) to test the effect on 5-HTTLPR, and results showed that participants carrying the S allele chose disadvantageous decks on the IGT task than L carriers and L carriers showed higher levels of loss aversion than S carriers. Finally, this paper makes a small contribution to the mapping of genes and behaviour. We do not know yet about the functionality of 5-HTTLPR in relation to other polymorphisms and genotypes. Further research is needed to explain how the serotonin system interacts with other genes and gender in order to draw a general conclusion on how the serotonin system influences human behaviour. Future studies should increase the number of polymorphisms with regard to impulsivity and perceived parenting; the dopamine receptor gene (DRD4 [36]), and the androgen receptor gene (AR [37]).

Despite these limitations, the present study shows some evidence that the 5-HTTLPR genotype influences impulsive behaviour, and that this influence depends on the environment in which the individual was raised, or at least on his/her perception of the environment in which he/she was raised. The seemingly infinite complexities of social behaviour, difficulties in “measuring” it, influence of environment, interactions with genes, and influence of epigenetic phenomena may be amongst the confounding factors that present an apparently insurmountable obstacle to understanding the causal links between genetics and social behaviour. To advance understanding of these complexities, further research needs to be applied in a more controlled way, e.g. matching experimental set-ups with population-based and clinical samples with a sufficient sample size.
